# Intraocular lenses in age-related macular degeneration

**DOI:** 10.1007/s00417-017-3740-8

**Published:** 2017-07-24

**Authors:** Andrzej Grzybowski, Weronika Wasinska-Borowiec, Jorge L. Alio, Pedro Amat-Peral, Juan Tabernero

**Affiliations:** 1 Chair of Ophthalmology, University of Warmia and Mazury, Olsztyn, Poland; 2 Department of Ophthalmology, Poznan City Hospital, Poznań, Poland; 3 ul. Szwajcarska 3, 61-285 Poznan, Poland; 4 Department of Ophthalmology, University of Warmia and Mazury, Olsztyn, Poland; 5 Vissum Corporation, Alicante, Spain; 6 Division of Ophthalmology, Universidad Miguel Hernández, Alicante, Spain; 7 Vision and Eye Research Unit, Anglia Ruskin University, Cambridge, UK

**Keywords:** Implantable miniature telescope, IOL-VIP system, Lipshitz macular implant, Fresnel prism intraocular lens, iolAMD, Scharioth macula lens

## Abstract

**Purpose:**

The aim of this work is to review the lenses, assessing their advantages and disadvantages. We describe a total of seven types of intraocular lenses (IOLs) recommended for age-related macular degeneration (AMD).

**Methods:**

We used the PubMed web platform to search for implantable devices in various stages of AMD. We searched for both prospective and retrospective studies and also case reports.

**Results:**

Clinical results in AMD patients have been described for a total of seven types of IOLs recommended for AMD: an implantable miniature telescope (IMT), IOL-VIP System, Lipshitz macular implant (LMI), sulcus-implanted Lipshitz macular implant, LMI-SI, Fresnel Prism Intraocular Lens, iolAMD and Scharioth Macula Lens.

**Conclusions:**

We conclude that to objectively ascertain the effectiveness and safety of these lenses, further independent clinical studies with longer follow-up data are necessary prior to the general use of these optical devices.

## Introduction

Age-related macular degeneration (AMD) is one of the most disabling diseases for visual quality. Although an estimated 80% of AMD patients have non-neovascular or atrophic AMD, the neovascular form is responsible for nearly 90% of the severe central visual acuity loss associated with AMD [1]. The condition in a dry form is caused by aging and thinning of the tissues of the macula. Exudative macular degeneration occurs when choroidal neovascularization (CNV) is present [1, 2].

Central scotomas appear in the final stage of macular degeneration. This does not usually affect the peripheral vision [1, 3]. Age-related macular degeneration has been described as the leading cause of legal blindness, affecting 10–13% of adults over 65 years of age in North America, Europe, Australia and, recently, Asia [2].

Visual rehabilitation with low vision magnifiers has been the principal method for helping these patients, some examples are: hand/stand magnifiers, spectacles, hand held telescopes, closed circuit televisions, and high-plus spectacles in conjunction with high-minus contact lenses to create a telescopic effect. Although these tools maybe effective for correcting overall visual functioning, they have several limitations. They are cumbersome to use and cosmetically burdensome [4].

For several years, specially designed intraocular implants have become a possible and attractive way to circumvent many of the problems faced in extraocular visual aids. The aim of this work is to review the lenses assessing their advantages and disadvantages [5].

## Materials and methods

We used the PubMed web platform to search for implantable devices in various stages of AMD. We looked for both prospective and retrospective studies and also case reports. Our key words were strictly connected to our subject of concern: iolAMD, Scharioth lens, Fresnel Prism, IOL-VIP System, implantable miniature telescope, Lipshitz macular implant. We selected English language articles strictly connected with intraocular lenses used in diagnosed AMD. Only lenses with peer reviewed, published clinical outcomes in human patients affected by AMD were considered for this review.

## Results

This article summarizes the mechanism and results for seven different IOLs designed to help patients with AMD: an implantable miniature telescope (IMT), IOL-VIP System, Lipshitz macular implant (LMI), sulcus-implanted Lipshitz macular implant, LMI-SI, Fresnel Prism Intraocular Lens, iolAMD and Scharioth Macula Lens. The summary of clinical results is presented in Table 1.

**Table 1:** Summary of the clinical data from selected articles

	IMT	WA IMT	IOL-VIP System	LMI LMI-SI	Fresnel Prism Intraocular Lens	iolAMD	Scharioth Macula Lens
Number of cases reported	3 [6] 40 [7]	217 [5]	40 eyes of 35 patients [8]	6 [9] 3 [10]	3 [11]	3 eyes of 2 patients [12] 18 eyes of 12 patients [13]	8 [14]
Mean preoperative visual acuity	0.15 with no external telescope [6] UDVA 0.1 UNVA 0.11 [7]	20/80–20/800 [5]	CDVA 1.28 Reading distance (cm) 4.44 [8]	DVA 1.47 NVA 24.16 [9] no data [10]	CDVA 0.12 [11]	CDVA 0.08 CNVA 0.03 or less [12] CDVA 0.12 CNVA <0.14 [13]	CDVA 0.05–0.4 CNVA @ 40 cm 0.18 CNVA @ 15 cm 0.3 [14]
Mean postoperative visual acuity	0.24 after 18 months [6] UDVA 0.2 UNVA 0.22 [7]	12 months post-op mean 3.43 lines improvement in BCDVA [5]	CDVA 0.77 Reading distance (cm) 12.18 [8]	DVA 0.94 NVA 75.00 [9] No data [10]	CDVA 0.1 [11]	CDVA 0.64 CNVA 0.64 [12] CDVA 0.20 CNDA 0.21 [13]	UNVA @ 15 cm 0.5 [14]
Gains in lines or letters	No data [6, 7]	After 60 months: 2.1 lines (>75 years old), 2.7 lines (younger patients) [5]	Reading distance gain - 7.66 cm [8]	3.66 lines change in ETDRS score 50.83 [9] no data [10]	No data, displacement of the scotoma (all patients) [11]	No data [12, 13]	CNVA @ 40 cm - 4.4 lines CNVA @ 15 cm - 2.1 lines [14]
Complications	Narrow field of view (all patients) [6] Pupillary cyclitic membrane, synechiae, posterior capsular opacification, corneal edema, hyphema, conjunctivitis, uveitis [7]	Corneal edema iris damage/ prolapse capsular rupture, removal of the device [5]	None [8]	Slight glare (all patients); shadowing of images that occluded the un-operated eye (2 patients); adapted within 3 months of surgery [9] No data [10]	Posterior capsule opacification (1 eye) [11]	Diplopia for distance vision (one patient, monocular implantation) [12] In 1 eye, an anterior sulcus IOL was replaced [13]	None [14]

*UDVA* uncorrected distance visual acuity, *UNVA* uncorrected near visual acuity, *CDVA* corrected distance visual acuity, *CNVA* corrected near visual acuity, *BCDVA*best corrected distance visual acuity, *upper index* number of reference

### Optical fundamentals of IOLs for AMD

The most common approach, used in IMT lenses, the IOL-VIP System, and iolAMD is a Galilean type telescope. For the Galilean approach, two optical elements with high positive and negative power should be used in combination with the cornea. IMT lenses can achieve higher magnification than IOL-VIP System and iolAMD because the positive and negative lenses are embedded in air. This configuration increases the dioptric power in each of the lenses in an order of magnitude that cannot be achieved with lenses embedded in an aqueous medium. On the other hand, it requires the implantation of a long tube through a larger corneal incision. The IOL-VIP System requires implantation of the positive lens in the anterior chamber and as with the iolAMD there is a version that incorporates a decentration of one of the IOLs to generate a displacement of the retinal image from a potentially damaged central retinal area. The iolAMD incorporates asphericity in the positive lens to gain depth of focus and to be highly tolerant to small changes in the nominal axial distance between both lenses. The lenses are smaller and thinner and the positive optical elements are implanted in the sulcus [6–9, 13–17].

Another telescope approach is the LMI, based on a Cassegrain configuration, which uses mirrors instead of lenses. It can provide high magnification, but due to the sophistication of the device it probably requires higher manufacturing costs, especially compared to a simple silicon or acrylic IOL. Additionally, the use of small mirrors might generate the risk of glare effects, due to diffraction and ghost reflections in the elements that should be further investigated in clinical trials [10, 11].

The Fresnel Prism Intraocular Lens provides no magnification at all. It only displaces the retinal image from a potentially damaged central macula to a more peripheral healthier area in the retina. The Fresnel approach (the partition of the optical surface in Fresnel zones) is necessary here to provide the required tilt of the image, as the introduction of a direct prism in the whole surface of the lens would not be possible in practice (one of the edges of the lens would be too thick). A potential problem of this approach might be diffraction and scattered light at the edges of each Fresnel zone. As shown in Fig. 5, there might be some light that is scattered away from the focal point, which might be a source of glare [12].

Finally, the approach used in the Scharioth Macula Lens is based on magnification at closer distances. The closer the object to the eye, the higher the magnification. In this approach it needs to be considered that the subject is unable to accommodate and for that reason it incorporates a +10 D central area in the lens. Magnification is only achieved when the object is in a range of 10 to 15 cm from the eye. It provides no distance vision magnification [18].

### Clinical outcomes

#### Intraocular magnifier telescope (IMT)

It should be emphasized that the short name of this lens is quite confusing. The “IMT” abbreviation is commonly used for all models of the implant. In fact, it should refer to the original model, which went out of use in 2001. After that, the production of a “wide-angle” model began, sometimes abbreviated as “WA IMT”. With high probability we can say that scientific publications after 2001, despite the abbreviation IMT, refer precisely to the “wide-angle” model, but unfortunately it is unclear. The “WA” prefix is used for the two product models available differing in magnification: a wide-angle 2.2X and wide-angle 2.7X. The first one gives full field of view of 24° and the second one, 20°. The other features are the same for both models.

For the purpose of this chapter, we are referring to the currently used version of the implant.

An implantable miniature telescope (IMT) prosthetic device was designed specifically for patients with the most advanced or end-stage form of AMD. The IMT combined with the optics of the cornea produces a telephoto effect that enlarges images in a patient’s central visual field 3× with a 20–24° field of view projected onto approximately 55° of the retina. Intended for monocular implantation, the implanted eye provides central vision, while the other eye remains “as is” to retain peripheral vision, which is important for maintaining balance and orientation. As it is implanted rather than being external and hand-held, the IMT allows patients to see in both dynamic and static situations at near, intermediate, and distance vision ranges [15, 19–21].

**Fig. 1:**
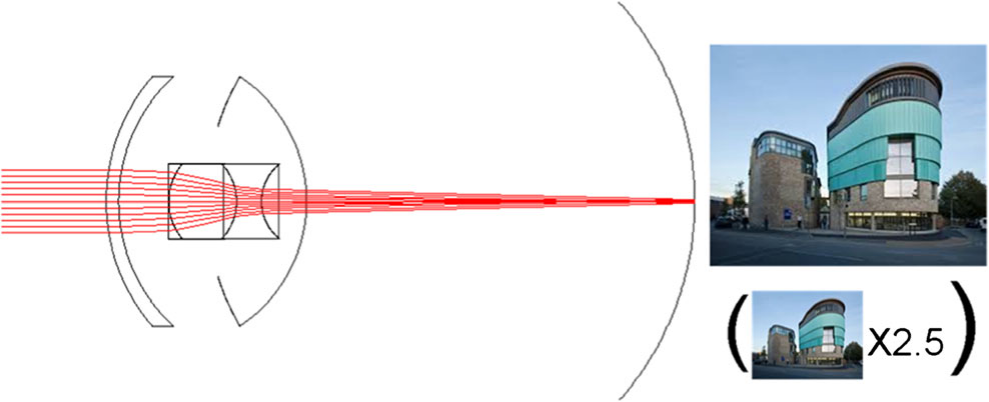
Manner of action of the Implantable Miniature Telescope (IMT)

The IMT is a fixed-focus quartz glass lens with wide-angle micro-optics which is implanted in the capsular bag through a 10–12 mm incision after the natural lens has been removed (Fig. 1). Larger than most implanted devices, the IMT is a 4.4 mm long telescope contained in a polymethyl methacrylate (PMMA) carrying device with an overall haptic-to-haptic diameter of 13.5 mm. Two modified C-loops facilitate in-the-bag fixation. The device weighs 60 mg in aqueous; 115 mg in air. The lens aperture is 3.2 mm. The IMT extends through the pupil and remains on average 2.5 mm from the posterior cornea, preventing damage to the endothelium [5, 16, 22, 23].

The US FDA first approved the device in June 2010, originally restricting implantation to patients older than 75 years of age. In 2014, the FDA lowered this criterion to 65. Suitable patients must also be phakic in at least one eye and meet other vision and eye health criteria [15].

The first systematic clinical report with the first generation device was published by Alió et al. They performed a multi-center study in which the IMT was implanted in 40 eyes of 40 patients with dry-type AMD. Patients were followed up for 12 months. They concluded that the IMT played an important role in improving near and far visual acuity in patients with stable dry-type AMD. However, they stressed the problem of severe visual field restriction and the cumbersome postoperative visual rehabilitation [7].

Due to the optical principle on which this IOL is based, it was not difficult to observe in the reports that a main disadvantage of IMT implantation is the confined central visual field to a 20° angle. The IMT is implanted in one eye only while the other eye is used to preserve the peripheral visual field, that is why binocularity is lost with this procedure [7] (Fig. 1).

The IMT is well documented in the literature. Two multi-year clinical studies have been conducted to evaluate the safety and efficacy of the telescope implant: the IMT-002 pivotal safety and efficacy study and the IMT-002-LTM long-term monitoring safety study [15, 19]. The 2-year, prospective, 28-center IMT-002 pivotal study (*n* = 217) evaluated the safety and effectiveness of the IMT for the improvement of visual acuity in patients with bilateral moderate to profound central vision impairment (best corrected distance visual acuity (BCDVA) between 20/80 and 20/800) due to untreatable end-stage AMD [24]. The IMT improved visual acuity and quality of life in subjects with end-stage AMD. The primary effectiveness endpoint, a 2-line or greater gain in either distance or near best corrected visual acuity (BCVA) at 12 months in at least 50% of study subjects was met and exceeded. Approximately 90% of patients demonstrated two or more lines of improvement on the ETDRS visual acuity chart and 67% of patients were able to see three or more lines after the surgery compared to 13% of fellow eye controls.

At the 12-month mark, an assessment using the National Eye Institute Visual Functioning Questionnaire-25 (VFQ-25) demonstrated that the telescope implant significantly improved quality of life in this study population. Not only was there an improvement in vision-specific subscales, which would be expected with a doubling of visual acuity achieved when participants were trained to understand their new vision post-implantation, but there was also a significant improvement in the psychosocial vision-targeted dependency, mental health, role difficulties, and social functioning subscales. Results suggest that patients are less dependent on others, less worried or frustrated with their visual acuity, less limited in their activities related to visual acuity, more able to visit others, and better able to recognize facial expressions [19].

Ocular complications encountered during the studies included endothelial cell loss, inflammatory/pigment deposits, transient cornea edema and IOP elevation. Regarding concerns about endothelial cell loss, it has been shown to be consistent with that reported for conventional IOLs [15, 25, 26].

It is also reported that three eyes required explantation of the lens because of patient dissatisfaction and a conventional posterior chamber IOL was implanted [7].

Patients participating in the company’s 24-month pivotal safety and efficacy study, IMT-002, were invited to participate in the IMT-002-LTM extension study. Patients were followed for 60 months after IMT implantation. Efficacy and safety data were analyzed for the entire patient population and were then further stratified by age for two patient cohorts, those aged 65 to 74 (Group 1) and those aged 75 and older (Group 2). Overall, both groups of patients demonstrated substantial visual acuity improvement and retention, although outcomes were somewhat more favorable in the younger patient group. In Group 1, mean BCDVA improvement from baseline was 3.3 lines at 24 months and 2.7 lines at 60 months. In Group 2, mean BCDVA improvement from baseline was 3.1 lines at 24 months and 2.1 lines at 60 months. A substantially larger percentage of patients in Group 1 retained three or more lines of vision at month 60 than in Group 2 (58% vs. 38%, respectively). Younger patients also had fewer reported adverse events than their older counterparts. The most frequent adverse events (AEs) in Group 1 were iris prolapse (*n* = 6/70; 8.6%) and iris damage (*n* = 4/70; 5.7%). The leading AEs in Group 2 were corneal edema within the first 30 days after surgery (*n* = 10/127; 7.9%) and iris transillumination defects within the first 30 days after surgery (*n* = 7/127; 5.5%). Group 2 reported more AEs in 11 of the 14 reported category events [15].

As described by Joondeph, telescope-implanted eyes may develop choroidal neovascularization because of the natural course of age-related macular degeneration, although it is rare and develops in less than 0.5% of eyes. He describes a technique in which these patients can be diagnosed via ocular coherence tomography imaging and treated with intra-vitreal injection, similar to phakic or pseudophakic eyes. In must be stressed that the patient’s commitment to the visual rehabilitation to adapt to their new field of vision in one eye combined with peripheral vision in the other eye is critical for the success of the telescope implant procedure. Low vision specialists are an integral part of the procedural team because they teach patients exercises related to static and dynamic movement [27].

The diagnosis and management of CNV requires optical coherence tomography (OCT) imaging of the macula, which presents several unique challenges to the physicians when performing it in patients with an IMT. The first obstacle is due to the IMT itself; it is difficult, if not impossible, to get a clear view of the macula through the IMT. Fundus photography or angiography through the IMT creates a minimized, distorted image. The second limitation is that patients with the IMT may be unable to focus on the fixation target during an OCT session due to the loss of central vision, resulting in constant scanning or moving of the eye. In addition, the longer scan time required due to the aforementioned poor fixation may lead to drying of the corneal surface, further impairing the view of the fundus [28, 29]. Some of the methods physicians may use to overcome these barriers to capture high-quality OCT images include lubrication of the ocular surface with artificial tears, the best possible dilation of the pupils, and using anatomical landmarks such as the optic nerve to locate the macular region [30, 31].

Some modifications in certain features of the OCT increase the likelihood of detecting retinal fluid in eyes with the IMT. For example in SD-OCT all retinal scans should use the 20° lens and the fast preset volume scan pattern. Condensing the area of the preset volume scan pattern overcomes the minimization effect of the telescope to focus on the macular region and improves the quality of the image. Changing the number of sections from 23 to 193 can achieve greater anatomic detail of the fovea [31].

#### IOL-VIP system

**Fig. 2:**
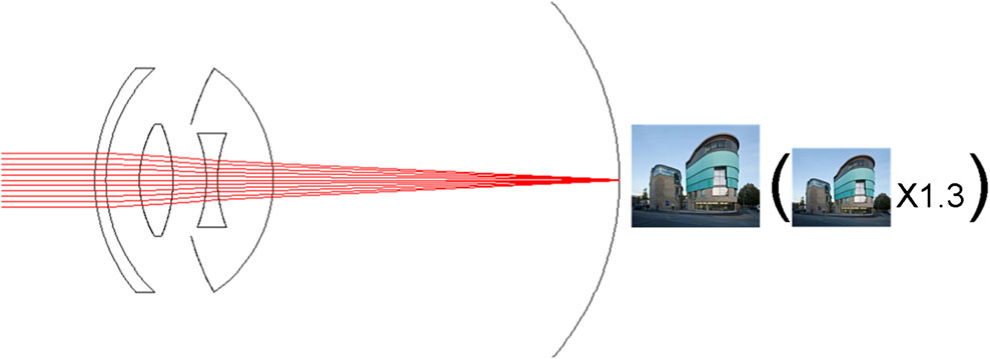
Manner of action of the IOL-VIP System

The IOL-VIP System consists of two IOLs that reproduce an intraocular Galilean telescope: a high minus-power biconcave IOL (about 66 diopters D) in the capsular bag acts as the eyepiece, and a high plus-power biconvex IOL (about 55 D) in the anterior chamber (AC) acts as the objective (Fig. 2). Both lenses are made of polymethyl methacrylate, have a one-piece design, and provide ultraviolet light filtering. The optic of the two lenses is 5 mm in diameter, with a maximum axial thickness of 1.5 mm for the AC IOL and peripheral thickness of 1.5 mm for the in-the-bag IOL; their total length is 13 mm. The system provides an estimated magnification for distance of 1.3 [5, 8, 9].

Insertion of the IOL-VIP System is preceded by a standard phacoemulsification. Due to the thickness of the IOLs, the surgical protocol recommends a capsulorrhexis with a diameter of at least 6 mm to facilitate the implantation of the in-the-bag IOL, with the enlargement of the temporal corneal incision to up to 7 mm [9].

The candidates for IOL-VIP System implantation are selected using software that collects their clinical data. All patients undergo a 2-week preoperative training (12 30-min training sessions) and a 3-month postoperative rehabilitation program (five 30-min training sessions per week for 12 weeks) aimed at training and consolidating the preferred retinal locus (PRL) [5, 9].

One report described the outcomes observed in forty eyes of thirty-five consecutive patients with a stable central scotoma due to macular disease who underwent phacoemulsification cataract surgery with the implant of the IOL-VIP System [9]. All patients showed an improvement in visual acuity (VA) due to the surgical and rehabilitative procedure, confirming or exceeding the preoperative expected results. Mean postoperative best corrected visual acuity (BCVA) was 0.77 (logarithm of the minimum angle of resolution), compared to 1.28 preoperatively. The mean postoperative best reading magnification gain was 6.2, and the mean postoperative reading distance gain was 7.66 cm. It was highlighted that the IOL-VIP System was subjectively well tolerated and did not seem to limit the peripheral visual field or interfere with binocular vision, thus making it suitable for monocular or binocular implantation. There were no severe complications intra or postoperatively with the exception of pupillary block with increased intraocular pressure, promptly managed by means of neodymium:yttrium–aluminum–garnet (Nd:YAG) laser iridotomy. Due to this, preoperative iridotomy was performed in all other cases [9].

Given the size of the two IOLs, there may be concerns about the space they occupy in the anterior segment of the eye and their proximity to critical ocular structures such as the corneal endothelium and iris. It is true that after implantation there may be a shallow anterior chamber, so an exceedingly low endothelial cell count, and/or guttata are obvious contraindications. However, 20-month observation showed that endothelial cell counts had decreased by only 7% at the end of follow-up [9] (Fig. 2).

#### Lipshitz macular implant (LMI)

**Fig. 3:**
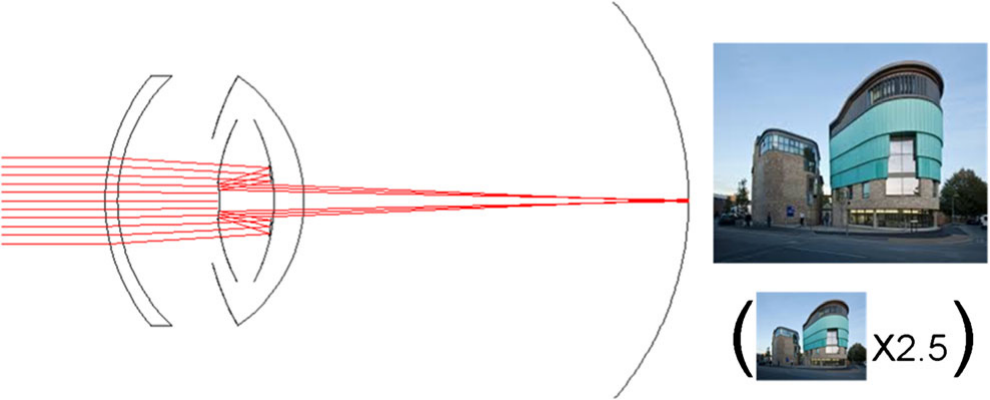
Manner of action of the Lipshitz macular implant (LMI)

An implant created by Dr. Lipshitz was made in two versions. The first was the Lipshitz macular implant (LMI) conventional IOL that incorporates two miniature mirrors in the Cassegrain telescope configuration, magnifying the reflected image on the retina 2.5 times. The patient thus sees a magnified central image through the mirror telescope and a normal non-magnified image through the periphery of the IOL [10] (Fig. 3).

The overall diameter of the IOL is 13.0 mm, and the optic is 6.5 mm. The anterior central mirror is 1.4 mm. The posterior mirror, which is doughnut shaped and 2.8 mm in diameter, has a central clear area of 1.4 mm in diameter. The peripheral zone of the optic is similar to that of a normal IOL in order to provide undisturbed peripheral vision. The reflecting surfaces of the LMI are coated with multiple layers of titanium oxide and silicon dioxide (dielectric coatings), which creates a mirror effect. The mirrors are 1 to 2 mm thick. The entire IOL is coated with poly-para-xylylenes (Parylene C) to enhance biocompatibility. The LMI is placed through a 6.5 mm corneal tunnel into the capsular bag [10].

It is reported that the LMI was implanted in six worse-seeing eyes of 6 patients. However only four of the operated eyes had AMD, two had other macular pathologies. In all patients visual acuity was worse than 20/200 and it improved with a 2.5 magnifying external telescope preoperatively. There were no intraoperative complications. The mean gain in distance acuity was 3.66 lines ± 1.88 (SD), and the mean increase in the Early Treatment Diabetic Retinopathy Study (ETDRS) score for near acuity was 50.83 ± 9.15 logMAR. The best corrected distance acuity and near acuity improved significantly (both *P* = .014) [10].

The younger and improved version of the LMI has the same function - to magnify the central image while the peripheral field remains normal. The main difference between the two lenses is that the newer LMI-SI is the equivalent of two IOLs and is well supported by placement within the capsular bag alone. The LMI-SI is a non-foldable one-piece IOL positioned in the sulcus over a regular bag-implanted IOL. It is 5 mm or 6 mm in diameter, and it contains loops that have a similar configuration to a regular IOL (loop diameter is 13.5 mm). However, the LMI-SI is thicker, with a central thickness of 1.25 mm. After standard phacoemulsification, the incision is then enlarged to 5 to 5.5 mm. After implantation a peripheral iridectomy is then performed surgically [11].

According to a publication, three patients were operated on using the LMI-SI IOL. The inclusion criteria for a pilot trial included patients with bilateral AMD (dry type, wet type, or scar stage) or other similar macular lesions in which visual acuity ranged between 20/80 and 20/800 in each eye and improved for distance and/or near when tested with 2.5 magnification using an external telescope. Postoperative visual acuity of these patients is not reported in the publication [11].

The LMI and LMI-SI provide magnified central images up to 2.5 times while maintaining the normal peripheral vision through the peripheral portion of the lens. Due to this, both can be implanted in both eyes of a patient [10, 11] (Fig. 3).

#### Fresnel Prism Intraocular Lens

**Fig. 4:**
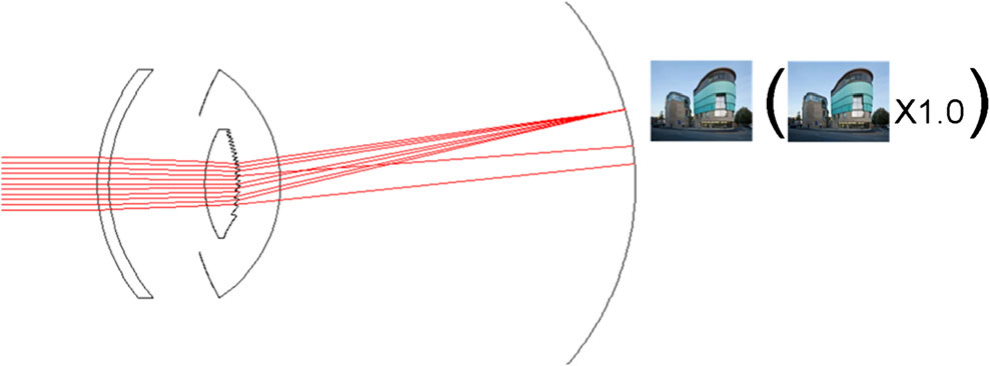
Manner of action of the Fresnel Prism Intraocular Lens

The in-the-bag Fresnel Prism Intraocular Lens is a non-foldable implant made of polymethyl methacrylate (PMMA) (Fig. 4). It was created for optical displacement of the central scotoma caused by AMD, to avoid moving the retina with all the risks involved in macular translocation surgery [12].

For implantation, standard phacoemulsification is performed and then a scleral tunnel incision is created for insertion. The prototypes of the device have a single optical power (+20.0 diopters) for aphakic correction, with a Fresnel prism IOL fashioned on the posterior surface of the optic producing a fixed 6° deviation, which gives a retinal image displacement of 1.8 mm (thus describing a circular area of 3.6 mm diameter) for a 23.1 mm average eye [12].

The only publication we found on the Fresnel Prism Intraocular Lens reports that the implant was fixed unilaterally in three eyes of three patients with bilateral advanced nonexudative AMD. The inclusion criteria were bilateral AMD, a decimal corrected distance visual acuity of 0.1 or worse in the better eye (eye for the IOL implantation), and a lesion diameter no larger than 3.3 mm. Preoperatively, direction of the image deviation was identified using a handheld visuoscope to identify the preferred retinal locus for extrafoveal fixation. The uncorrected distance visual acuity (UDVA) of all patients was 0.05 and corrected distance visual acuity (CVDA) was between 0.05 and 0.16. After the surgery, no objective testing of scotoma displacement was performed; however, all patients reported displacement of the scotoma peripheral to their central field of vision and noted that the scotoma was less bothersome. No patient described diplopia. One patient reported that she preferred her un-operated eye to the eye with the prismatic IOL, despite having a less obvious eccentric scotoma in the operated eye. This was because she perceived the image to be clearer in the un-operated eye. Postoperatively, UDVA in the three eyes was between 0.05 and 0.10 and CVDA 0.05–0.16 [12].

Oculomotor functions and fixation stability control change in relation to the new preferred retinal fixation locus. The newly formed oculomotor functions can be improved by exercising or by spectacle prismatic image relocation [12] (Fig. 4).

#### iolAMD

**Fig. 5:**
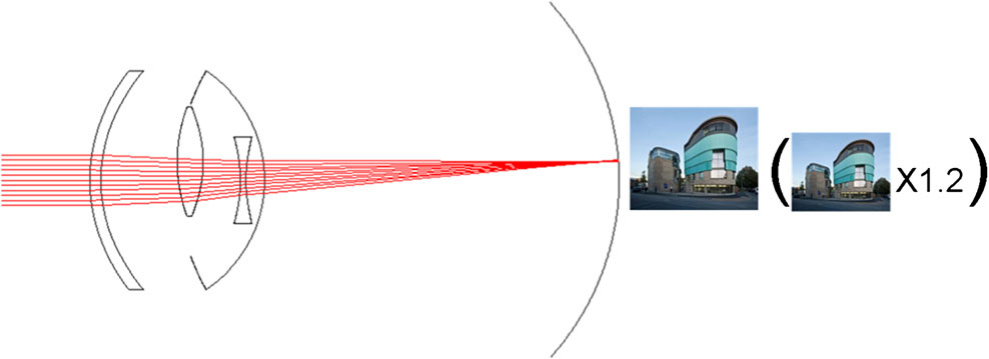
Manner of action of the IOL AMD

The iolAMD is the most recent type of hydrophobic acrylic device to improve vision for people suffering from AMD. The implant is based on a Galilean telescope using two lenses manufactured so that they can be injected with a standard soft tip cartridge and injector system for 3.0-mm incision size (Fig. 5). After implantation, both implants allow a magnification of the image and distribution of the retinal picture 3° apart from the fovea due to the slightly intended decentration of the sulcus implanted IOL (0.85 mm). The capsular bag positioned IOL (IOL 2) is a high-minus-power lens (−49 diopters [D]) with a 4.0-mm optic and an overall length of 11.0 mm. The plate haptic is symmetrical and vaulted posteriorly approximately 15°. The sulcus-positioned IOL (IOL 1) is a high-plus-power lens (+63 D), and the 5.0-mm hyper-aspheric-optic is slightly de-centered on the plate haptic. The overall diameter is 11.75 to 12.0 mm and the haptic is bent anteriorly to enhance the recommended distance between the optics of 2 mm after implantation [13, 14, 17].

It is reported that three eyes of two patients with visually significant cataract and intermediate dry macular degeneration with drusen were operated by phacoemulsification with insertion of the iolAMD. Preoperative corrected distance visual acuity ranged from 0.03 to 0.16 and corrected near visual acuity was 0.03 or less. No surgeries had any complications. There was no rise in intraocular pressure or iris-related problems such as shaving of pigment or pupillary block. The patient with bilateral implantation perceived no double vision because the decentration axis in both eyes was vertical. The other patient with singular implantation recognized an increase in visual acuity but complained about diplopia in a vertical direction (which could be solved by prismatic spectacle correction). All lenses were placed on a vertical axis and the IOL implantations were achieved safely and were stable during the 3-month follow-up. No postoperative rotation was necessary. Two months after surgery, corrected distance and near visual acuities increased to levels between 0.5 and 0.8 (uncorrected distance visual acuity was 0.3 to 0.6; uncorrected near visual acuity was 0.1 to 0.8) [17].

In another study, 18 eyes of 12 patients had the iolAMD implanted. Inclusion criteria included bilateral intermediate or advanced dry AMD with central scotomata; minimal cataract or pseudophakia; Snellen corrected distance visual acuity (CDVA) of less than 0.25, improvement on simulation with the new injectable telescopic IOL (CDVA or subjective improvement). All surgeries were uneventful except in one eye in which the high-plus IOL was vaulting anteriorly, causing a reduction in the quality of vision. This high-plus IOL was replaced with a smaller-diameter IOL, after which there were no short to medium term sequelae. A precautionary intraoperative peripheral iridectomy was performed in nine eyes (including the eye of patient 11 in which the high-plus IOL was replaced). In eight eyes, a single 10-0 nylon suture was used to secure the wound as a precaution; the suture was removed 1 month after surgery. There was no difference between the mean preoperative and postoperative intraocular pressure. The mean endothelial cell density was reduced by 18%. The mean decimal CDVA improved from 0.12 preoperatively to 0.20 at 4 months, a 67% gain [14].

The only noticeable disadvantage of this system is that there are no power ranges available, limiting this technology to eyes with an axial length of 21 to 23 mm with a resulting power of 21 D. The IOL power cannot currently be adapted perfectly to patient anatomy [17] (Fig. 5).

#### Scharioth Macula Lens

**Fig. 6:**
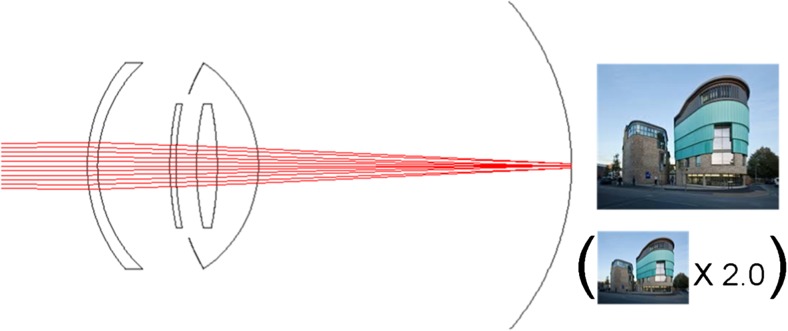
Manner of action of the Scharioth Macula Lens

The Scharioth Macula Lens is a one-piece foldable intraocular hydrophilic acrylic lens with a central magnifying portion implanted in the ciliary sulcus of pseudophakic eyes, which improves near vision in patients with AMD (Fig. 6). The overall diameter of the IOL is 13.0 mm with four symmetric haptics. It has a central portion of 1.5 mm diameter with an addition of +10.0 Dsph and neutral remaining optical zone [18].

The IOL can be implanted simultaneously during uncomplicated standard phacoemulsification with in-the-bag posterior chamber IOL (PC IOL) implantation or years after cataract surgery, making it unique among other IOLs implanted only during a procedure to remove a clouded lens. Another exceptional feature of this device is the smallest incision required for implantation -2.2 mm. The macular add-on IOL does not affect the peripheral vision and does not reduce binocularity at normal reading distance. Binocularity is reduced only at a reading distance of 15 cm. At this distance, the image of the other eye will be blurry and will not cause diplopia [18].

A minimum corrected distance visual acuity (CDVA) of 0.1 is recommended to achieve sufficient results. Preoperative testing of corrected near visual acuity (CNVA) with +2.5 D correction at 40 cm versus +6.0 D correction at 15 cm provides valid information about the potential of the macular add-on IOL; if CNVA is better at 15 cm and the patient is motivated, he or she might be a good candidate for Scharioth Macula Lens implantation. Possible contraindications to the implantation of the macular add-on IOL are: complicated cataract surgery (e.g., aphakia, sulcus implanted posterior capsule IOL), excessive zonular weakness (e.g., excessive pseudoexfoliation syndrome, zonular dialysis, pseudo-phacodonesis), excessive secondary cataract, chronic uveitis, active rubeosis iridis, central corneal opacities, and inability to understand the principle of this implant (reduced reading distance, maximum magnification) [18].

It is reported that the macular add-on IOL was implanted in the better seeing eye in eight patients. The preoperative CDVA in the patients was between 0.05 and 0.4. No intraoperative or postoperative complications occurred. In all patients but one, the uncorrected near visual acuity (UNVA) at 15 cm and CNVA improved. The patient without improvement had a large area of retinal pigment epithelial atrophy of the posterior pole and a preoperative CDVA of 0.05. Excluding the eye with exudative AMD, the results were: CNVA improved by 5.0 lines with the macular add-on IOL at 15 cm versus with +2.5 D correction at 40 cm; it improved by 2.4 lines with the macular add-on IOL at 15 cm versus with +6.0 D correction at 15 cm. No patient had a postoperative decrease in CDVA [18] (Fig. 6).

## Discussion

### Limitations and complications of IOLs for AMD

#### Incision

The first striking advantage of some of the IOL implantation is that it is a simple procedure that can be performed by all surgeons trained in phacoemulsification surgery. Devices like the Scharioth Macula Lens or the iolAMD need incisions no wider than 3.0 mm, while the IMT needs 10–12 mm incisions. The large incision could cause increased corneal astigmatism and the risk of further complications [3]. In the size of incision it is decisive whether the lens is foldable and this determines the material of which it is made - both the Scharioth Macula Lens and the iolAMD are acrylic [17, 18].

#### Pseudophakic eye

Since most lenses or their parts are implanted into a capsular bag, the result is that they are not appropriate for pseudophakic patients. The only two implants, which seem to meet these criteria are the Scharioth Macula Lens and the LMI-SI; the macular add-ons to a standard IOL have the advantage that they can be easily implanted in the ciliary sulcus years after cataract surgery without the need to explant the in-the-bag IOL or directly after standard phacoemulsification with regular bag-implanted IOL [11, 18].

#### Compulsory iridotomy/iridectomy

In some cases during the days after surgery a pupillary block developed with increased intraocular pressure and a pre- or intraoperative peripheral iridotomy/ iridectomy should be performed with the implantation of the IOL-VIP System, the IMT and the LMI-SI [9, 11, 15]. In the latter, Nd:YAG laser iridotomy is not recommended [11].

#### Capsulotomy

Treatment with neodymium:YAG laser is possible in such cases, but the laser beam should be directed through the clear part of the haptic and not the glass optic in the case of the IMT. The posterior part of the IMT presses against the posterior capsule, decreasing the incidence of posterior capsule opacification [7].

#### Fundoscopy

An important drawback of some types of lenses such as the IMT is that fundus examination is difficult or impossible. Although the IMT allows fundoscopy, the magnification is not sufficient to evaluate microscopic changes in the fovea (eg. progression of AMD) or to detect possible postoperative posterior segment complications of cataract surgery such as cystoid macular edema or retinal detachment. In the case of a sudden and profound decrease in visual acuity, only ultrasonography provides clues to the cause [16].

In addition, the LMI shows difficulty in seeing the ora serrata due to glare. A good central fundus view is possible around the mirrors but fundus photographs taken from the center of the lens for patients with implanted LMI had reflections from the posterior mirror [10].

#### Rehabilitation

One of the patients’ expectations associated to lens implantation is immediate improvement of vision and a quick return to life as prior to surgery. Unfortunately, lenses such as the IOL-VIP System require complicated visual rehabilitation. There is a special IOL-VIP software which designs the rehabilitation strategies based on preoperative and postoperative training of the preferred retinal locus. All patients undergo 2 weeks of preoperative training (12 30-min training sessions) and a 3-month postoperative rehabilitation program (5 30-min training sessions per week for 12 weeks) aimed at training and consolidating preferred retinal locus. Cases of unstable and peripheral preferred retinal locus have been reported with large search movements that did not change with the rehabilitation training [9].

Some authors also report that patients after IMT implantation require intensive training postoperatively and it takes 3–6 months, but there is no information about the type of this rehabilitation. It should be performed by trained low vision specialists [7, 15, 16].

#### Visual field

The serious drawback with some of the lenses like the IMT, is that magnification at both long and short distances is achieved but at the expense of a reduction in the visual field and depth of focus [13, 17]. Thus, bilateral implantation is not possible. The device is implanted in one eye only, leaving the fellow eye to compensate for peripheral vision [9, 15].

On the contrary, macular add-on IOLs do not affect the peripheral vision and do not reduce binocularity at normal reading distance [18].

## Conclusions

In our opinion, there is no single ideal lens for use in existing AMD without drawbacks. The outcomes reported so far are variable and most probably have only been focused on short-term outcomes. The main problems found in the use of this technology are the strict patient selection criteria required to avoid quick evaluative forms of AMD and the need to choose eyes with a potential for visual rehabilitation. It is very important to emphasize that these patients need visual rehabilitation programs and that much of the success will depend on the commitment and dedication of the patient towards these programs. An important commercial bias may be present, however, in the reports of some of the iolAMD models due to a possible conflict of interest because of financial relations with the companies producing these lenses or the owners of patent rights. We may conclude that to objectively ascertain the effectiveness and safety of these lenses, further independent clinical studies with longer follow up data are necessary prior to the general use of these optical devices.

All figures are in the same scale. Axial and transversal distances of the devices are the ones provided by the manufacturers. The lens capsule is 4 mm length, used for reference here. The model eye used here had a length of 23.5 mm.
